# Deep learning for cerebral angiography segmentation from non-contrast computed tomography

**DOI:** 10.1371/journal.pone.0237092

**Published:** 2020-07-31

**Authors:** Michał Klimont, Agnieszka Oronowicz-Jaśkowiak, Mateusz Flieger, Jacek Rzeszutek, Robert Juszkat, Katarzyna Jończyk-Potoczna

**Affiliations:** 1 Department of Radiology, Poznań University of Medical Sciences, Poznań, Poland; 2 Fast-Radiology, Poland; 3 1st Department of Radiology, National Institute of Oncology, Warsaw, Poland; 4 Department of Paediatric Radiology, Poznań University of Medical Sciences, Poznań, Poland; Banner Alzheimer's Institute, UNITED STATES

## Abstract

Cerebral computed tomography angiography is a widely available imaging technique that helps in the diagnosis of vascular pathologies. Contrast administration is needed to accurately assess the arteries. On non-contrast computed tomography, arteries are hardly distinguishable from the brain tissue, therefore, radiologists do not consider this imaging modality appropriate for the evaluation of vascular pathologies. There are known contraindications to administering iodinated contrast media, and in these cases, the patient has to undergo another examination to visualize cerebral arteries, such as magnetic resonance angiography. Deep learning for image segmentation has proven to perform well on medical data for a variety of tasks. The aim of this research was to apply deep learning methods to segment cerebral arteries on non-contrast computed tomography scans and consequently, generate angiographies without the need for contrast administration. The dataset for this research included 131 patients who underwent brain non-contrast computed tomography directly followed by computed tomography with contrast administration. Then, the segmentations of arteries were generated and aligned with non-contrast computed tomography scans. A deep learning model based on the U-net architecture was trained to perform the segmentation of blood vessels on non-contrast computed tomography. An evaluation was performed on separate test data, as well as using cross-validation, reaching Dice coefficients of 0.638 and 0.673, respectively. This study proves that deep learning methods can be leveraged to quickly solve problems that are difficult and time-consuming for a human observer, therefore providing physicians with additional information on the patient. To encourage the further development of similar tools, all code used for this research is publicly available.

## Introduction

Angiography is a medical imaging technique that can be used to visualize blood vessels in the body. As it is the lumen of the vessels that is visualized, a variety of pathologies that cause the narrowing or bulging of a vessel can be detected, including atherosclerotic disease, occlusions, aneurysms, and vascular malformations [1].

Cerebral angiography is nowadays performed using three different imaging modalities: computed tomography angiography (CTA), magnetic resonance angiography (MRA), and digital subtraction angiography (DSA). With much less adverse effects than the other methods, MRA (specifically, TOF MRA) is becoming the first choice for many patients. However, long acquisition time and high operational cost currently make it impossible to use it as the sole method of cerebral arteries visualization [2]. What is more, MRA may be contraindicated in patients with some types of metallic implants [3]. CTA and DSA are both associated with patient X-ray exposure and the application of intravenous contrast, however, the effective radiation dose for CTA is reported to be approximately one-fifth of the DSA dose [4].

There are some serious risks associated with the use of iodinated contrast media (ICM), including allergic reactions, contrast-induced nephropathy, and thyrotoxicosis [5]. Caution should be exerted when administering ICM to patients with renal failure, thyroid disease, and metformin use [6]. What is more, ICM administration can be associated with adverse reactions in some patients, including rare cases of death [7]. There are multiple approaches to how to reduce those risks. Recently, Park et al. [8] showed that ICM dose and injection speed reduction leads to lower rates of acute hypersensitivity reactions. Alternatively, it would be useful to develop a tool that enables to visualize blood vessels on non-contrast CT (NCCT) scans to entirely avoid contrast administration.

The aim of the study was to train a convolutional neural network using pairs of CTA and NCCT head scans so that the network would later be able to identify and segment cerebral vasculature on NCCT and generate a synthetic cerebral angiography.

## Materials and methods

### Patients

The study was retrospective. All the patients consented to the CT examination with contrast administration. The study was approved by the Institutional Review Board, with a waiver of informed consent for reviewing medical records and images of the patients. Full name of the ethics committee: Bioethics Committee of the Poznan University of Medical Sciences, Bukowska 70 Street, room A204, 60–812 Poznan, Poland. The initial study group included 153 patients who underwent brain NCCT examination directly followed by CTA at the University Hospital of Lord’s Transfiguration in Poznań between January 2018 and April 2019. 22 patients were excluded due to unsatisfactory imaging quality, e.g. low contrast saturation of the vessels or significant artefacts caused by stents or coils.

With the purpose of providing diverse data for the algorithm, cases with vascular abnormalities were also included in the training data. Intracranial aneurysms were the most frequent pathologies, comprising 45% of all cases (60 patients). Artery fenestration was present in three cases. Apart from vascular pathologies, brain ischaemia was observed in six cases, and one patient with ventricular system asymmetry was included.

**Fig 1:**
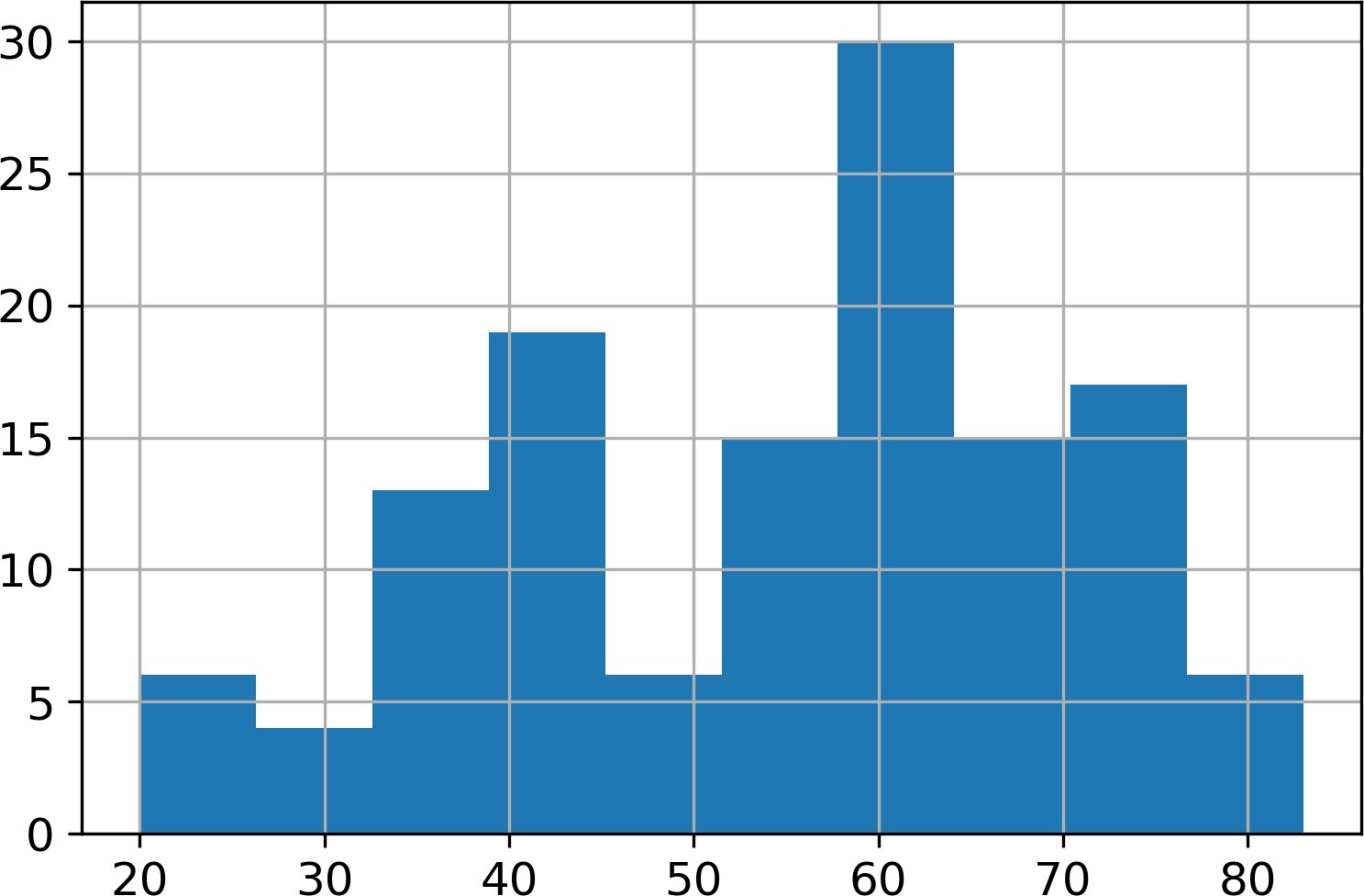
Histogram of patients’ age.

The final database included 131 pairs of NCCT and CTA scans of 131 different patients. The scans were stored as uncompressed DICOM files. All the scans were acquired using the GE Revolution Discovery CT/HD scanner. Water-soluble x-ray contrast agent with 370 mg/ml iodine concentration in the amount of 65–70 ml per patient was used with an injection speed of 4.5 ml/s. There were 87 females and 44 males in the study group. The mean age was 55.0 years (SD = 15.4, range 20–83), the mean age for female patients was 56.4 and the mean age for male patients was 52.3. The age distribution is presented in Fig 1. The imbalance between the genders of the patients was a direct result of more women having been examined in that period.

### Data preparation

Before training the network, the data underwent preparation. First, 3D Slicer 4.10.2 [9], an open-source software platform for the analysis and visualization of medical images, was used for the registration of NCCT images and CTA. Image registration can be defined as a process of spatially aligning two or more images [10]. To train the model, segmentation of the vessels from CTA along with registered NCCT was needed. In this case, the goal was to identify blood vessels on NCCT images by first segmenting them on registered CTA. The main issue when trying to segment vessels filled with contrast agent is the intensity overlap that occurs between vessels and bones. In CT, radiodensity values for bone and cartilage extend from around 200 to 3000 HU (Hounsfield Units), whereas the contrast enhanced blood vessels measure approximately between 120 and 500 HU. In this study, the areas with a density higher than 120 HU were isolated on both NCCT and CTA. This resulted in binary masks, with the mask generated from NCCT containing only bones, and the mask generated from the CTA containing both bones and vessels. The binary mask of NCCT was subsequently subtracted from the CTA mask, which resulted in segmentations encompassing only parts of the scans that contained contrast agent (i.e., vessels). This mask was further de-noised by removing small voxel areas of the mask that were isolated from other similar areas, which eliminated the remaining bone artefacts and produced a binary blood vessel mask for each patient.

**Fig 2:**
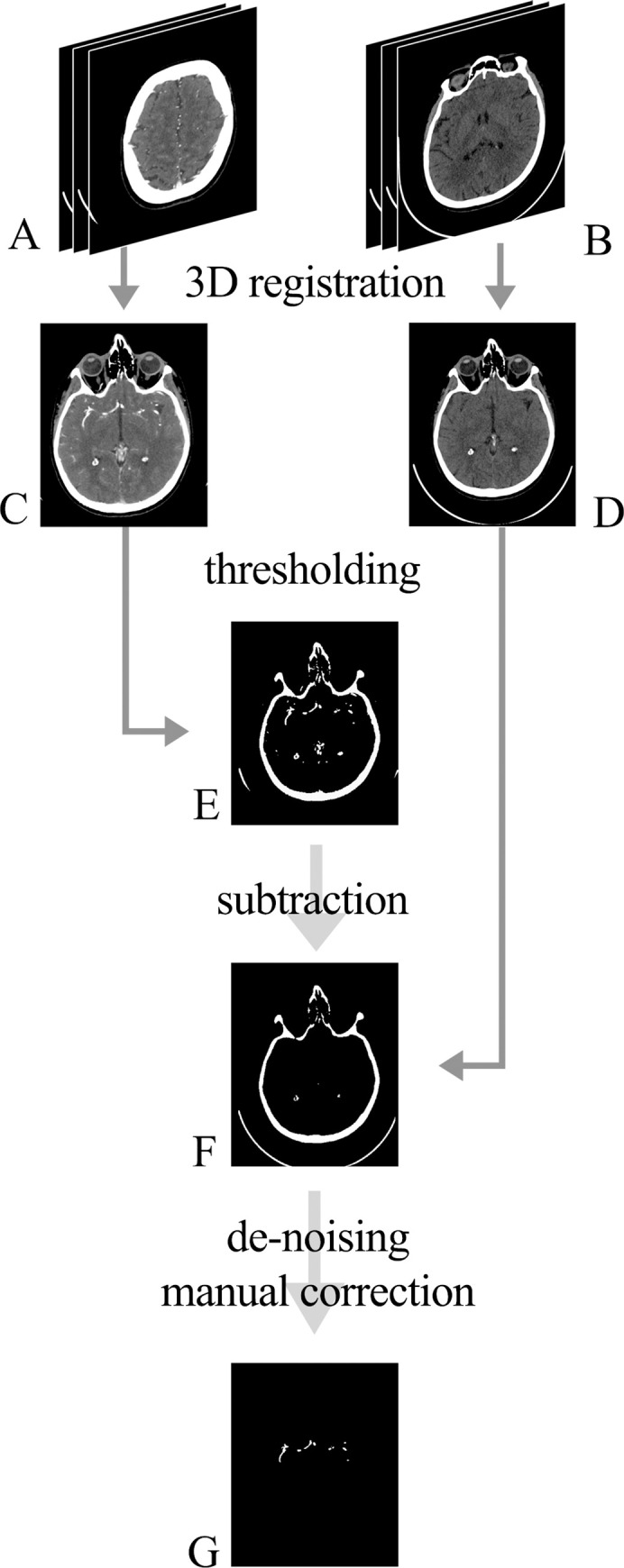
Data preparation. Raw DICOM images were obtained from the PACS system: A (CTA images) and B (NCCT images). Images were spatially aligned (registered) so that each pair of the 2D slices represented the same region (C and D). Using thresholding by radiodensity, binary masks were acquired from C and D, producing masks E and F, respectively. Subtraction of the mask F from the mask E was performed with further de-noising and manual refinement, which resulted in the final 3D segmentation of the vessels (G).

Finally, two radiology residents with two years of experience in reviewing CTA reviewed the binary masks using 3D Slicer. At this point, it was decided to manually remove dural venous sinuses, which were contrasted in some, but not all of the patients, as well as external carotid arteries with its branches, to provide homogenous data to the network. Fig 2 shows all the data preparation steps.

### Data preprocessing

Two approaches for data preprocessing were used. The first approach, **simple preprocessing**, involved clipping the values to a selected radiodensity range and then scaling these values to a float ranging from 0.0 to 1.0. As the neural network was pre-trained (see *Algorithm* section) on images containing three input channels, red, green and blue, and CT scans only contain one radiodensity channel, all the three channels had to be populated with data. Two configurations of this approach were tested. The first configuration consisted of applying the same radiodensity range, from -100 HU to 300 HU, and copying the obtained scaled values throughout all three input channels. The second configuration consisted of using different radiodensity ranges over each of the three channels: from -40HU to 120 HU, from -100 HU to 300 HU, and from 300 HU to 2000 HU. It was hypothesised that this could provide the algorithm with more information and enable it to generate more robust segmentations.

The second approach for data preprocessing, **uniform preprocessing**, involved converting the original bimodal distribution of radiodensity to resemble uniform distribution. This was achieved by dividing the radiodensity values (measured in HU) into 100 percentile bins of a similar size. Subsequently, a non-linear function was derived from the binning, which enabled scaling array values in DICOM images to a float tensor with values ranging from 0 to 1. Those values were copied over the three channels of the input data. This approach was also tested in two configurations. In the first configuration, the entire radiodensity range was used, and in the second configuration, the radiodensity range was clipped from -100 HU to 300 HU.

### Algorithm

U-Net architecture (a type of a convolutional neural network) has been used for this research following an original implementation by Ronnenberger et al. [11]. U-Net has been one of the most popular architectures in the medical imaging community for segmentation tasks, frequently outperforming previous state-of-the-art methods [11]. The network consists of two components: the encoder and the decoder, which are interconnected with the so-called skip connections. The encoder gradually reduces the intermediate resolution, i.e., downsamples it. Usually, this is performed by either striding [12] or pooling [13]. The decoder, on the other hand, gradually increases the resolution, i.e., upsamples it. Pixel shuffle with subpixel convolutional initialization was used during upsampling, as it is known to reduce checkerboard artefacts [14]. ResNet18 [15] has been used as the architecture for the encoder. The decoder consisted of mirroring layers, with downsampling replaced with upsampling [15]. To improve the modelling of long-range, multi-level dependencies across the image, self-attention mechanism was used on the top of the convolutional layer. It is known that training a deep architecture de novo requires thousands of entities [16]. To accelerate this process, the encoder was initialized with the weights trained on ImageNet dataset, which allowed the initial network to recognise basic shapes, such as edges and geometric shapes [17]. This approach is called transfer learning and is particularly useful for datasets of a limited size. For this reason, it has been widely used in the medical field, as it is sometimes impossible to acquire large datasets [18]. During the training phase, to increase the variability of the data fed to the network, random transformations were applied (Table 1). Non-medical datasets consist of images with RGB channels, therefore to make use of transfer learning, inputs to the network needed to have three channels as well. As it has been explained in the “Data preprocessing” section, two different approaches concerning the three channels were applied. Fastai library [19] has been used for training, validation, and testing. The neural network was trained in batches of 20 images (512x512 pixels). The values of the hyperparameters were selected on an empirical basis. For the training of the neural network, the novel “1cycle” learning rate policy was used, which allows to speed up training by decreasing the number of epochs and increasing the learning rate [20]. To optimize the parameters of the model, the Adam optimizer [21] with generalised Dice loss function [22] was used.

**Table 1:** Parameters of the model and image augmentation details.

**Network architecture**		**Training**	
Encoder	*ResNet18*	Learning rate	*1e-4*
Image size	*512 x 512*	Number of epochs	*16*
Self-attention	*True*	Batch size	*20*
		Weight decay	*1e-2*
**Image transformation**		**Optimization**	
Horizontal image flipping	*0*.*5 probability of occurrence*	Optimizer	*Adam*
Image rotation	*10° bidirectional with 0*.*75 probability of occurrence*	Learning rate policy	*1cycle*
Image zoom	*From 1*.*0 up to 1*.*2 with 0*.*75 probability of occurrence*	Loss	*Generalised Dice loss*

Neural network hyperparameters and image transformations are listed in Table 1.

To provide a baseline for the algorithm’s performance evaluation, a non pretrained model based on the original U-Net architecture was used [11]. Preprocessing remained the same for both methods. Both one-channel and three-channel approaches were tested.

### Evaluation

The data was split into two datasets. The first one contained 120 CT scans and was used for cross-validation, and the second one consisted of the remaining 11 scans and was used as a test set. The test set was isolated during the whole process of training and choosing the best model. The test set was used only on the final model that yielded the best results in the process of cross-validation.

The 120 patients dataset was used for 10-fold cross-validation, and groups (folds) of 12 patients were randomly sampled. During each fold, the algorithm’s performance was evaluated on one of the groups, while all the other groups were used as the model’s training set. For each of the folds, evaluation metrics were computed. Differences in model’s performance between the folds were analysed to assess the model’s performance consistency.

As an evaluation metric, the Dice coefficient [19] was used to analyse the similarity between the output (predicted angiography) and the ground truth (reference CTA).

Dice coefficient is a statistic used to measure the similarity between two samples for a binary target [19]. The formula for the Dice coefficient is as follows [19]: $\frac{2*TP}{2*TP+FP+FN}$.

Where TP, FP and FN are the numbers of true positives, false positives and false negatives, respectively. The Dice coefficient takes values between 0 and 1, with 1 meaning that two samples are identical, and 0 meaning that there are no common true positives in the two samples.

It was decided to compute the Dice coefficient for the entire CT examination and not for individual scans, as cerebral angiography is clinically assessed as a whole. For each fold, mean, standard deviation, minimum and maximum of Dice coefficient were calculated.

## Results

Each data preprocessing method and algorithm architecture were evaluated using cross-validation. The results can be found in Table 2. The simple preprocessing approach in the multiple windows configuration was characterized by the highest mean and was thus selected as the best performing one. It is worth noting that this approach was also faster than uniform preprocessing, as it requires fewer computations.

**Table 2:** Summary of the results of the cross-validation for the different preprocessing methods.

Preprocessing approach	Configuration	Dice coefficient			
		Mean	SD	min	max
Baseline U-Net without pretraining	One channel radiodensity range from -100 HU to 300 HU	0.656933	0.057193	0.429593	0.760778
	Three channels with radiodensity range: (-40, 120), (-100, 300), (300, 2000) HU	0.660420	0.056479	0.440012	0.765388
Simple	Radiodensity range from -100 HU to 300 HU on all 3 channels	0.669461	0.058315	0.433494	0.760348
	Radiodensity range: (-40, 120), (-100, 300), (300, 2000) HU	**0.673024**	**0.059828**	**0.432540**	**0.780889**
Uniform	Without clipping radiodensity range	0.664736	0.060725	0.424483	0.772273
	Radiodensity range from -100 HU to 300 HU	0.669326	0.061704	0.424889	0.772114

For each of the folds, the Dice coefficient was computed by calculating the mean Dice coefficient for individual patients within that fold.

After selecting the best data preprocessing approach and configuration, the model was trained on the 120 scans from the cross-validation set and evaluated on the test set. The mean Dice coefficient for this configuration on the test set was 0.638228 (SD = 0.080127, min = 0.467068, max = 0.720970). After identifying and excluding one outlier the mean Dice coefficient for the test set was 0.655344 (SD = 0.059607, min = 0.510662, max = 0.720970).

**Fig 3:**
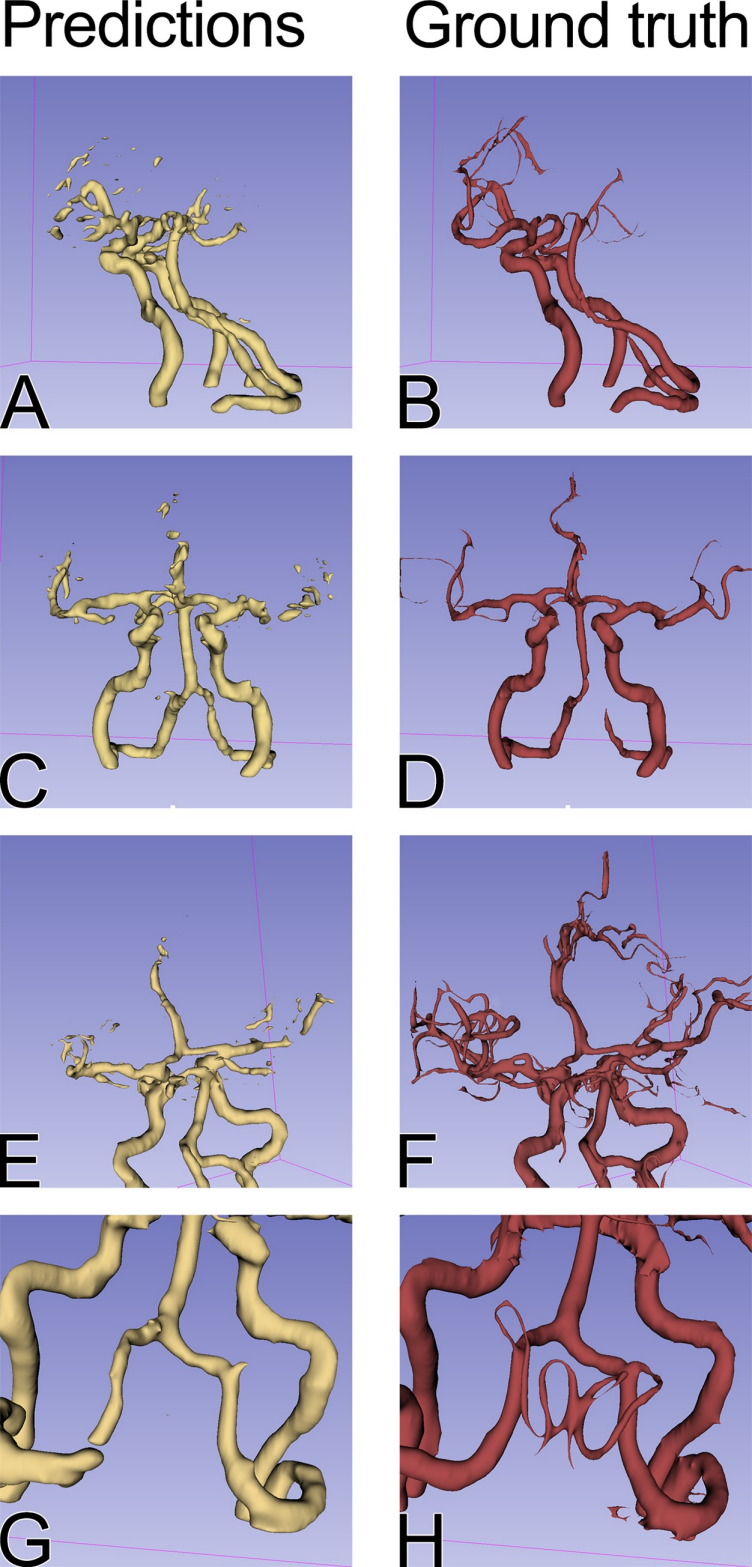
Comparison of the model predictions (first column, in yellow) and the ground truth (second column, in red) for the test set. Arteries of greater diameter, such as internal carotid arteries, middle cerebral arteries, vertebral arteries and basilar arteries, were identified by the model with high accuracy across the test set. The segmentation of the smaller arteries was more difficult on the branches of the middle cerebral artery beyond M2 segment (E and F). G and H compare the posterior inferior cerebellar arteries (PICA) segmentation and the ground truth. The model was able to identify only the proximal part of the left PICA.

**Fig 4:**
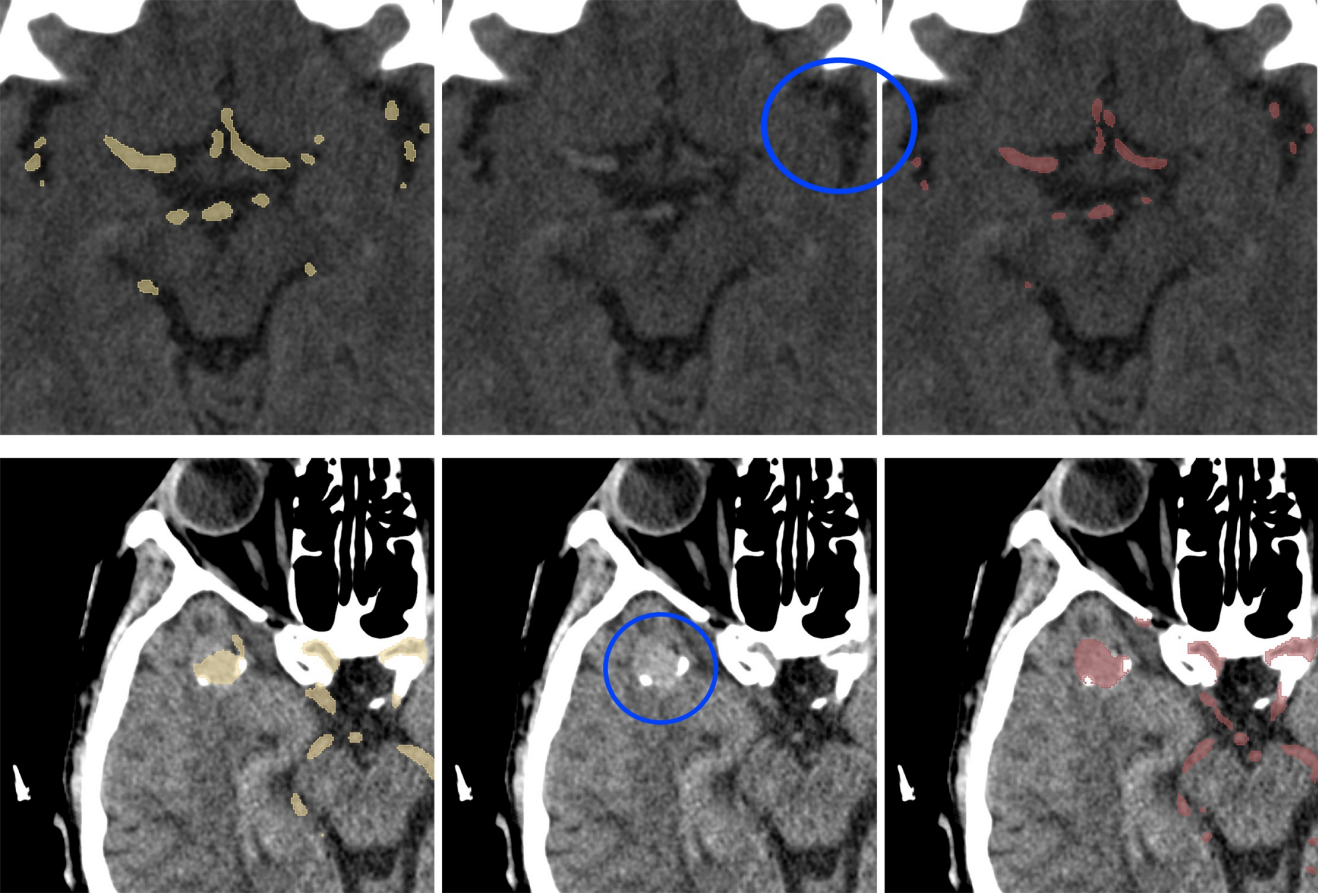
Test set cases, predictions and ground truth comparison. Model predictions (first column, in yellow), a corresponding slice of NCCT (second column), ground truth (third column, in red). First row: small arteries in the proximity of insular cortex (blue circle)—for a human observer, these are indistinguishable from noise. Second row: a large intracranial aneurysm (blue circle) and its segmentation.

Examples of the segmentations are depicted in Figs 3 and 4.

## Discussion

### Our results compared with other studies in the field

This study consisted of training a U-Net CNN on pairs of CTA and NCCT images to generate a synthetic cerebral angiography from NCCT brain scans. As far as we know, this is the first study of this kind. There were some attempts to generate synthetic contrast-enhanced images from non-contrast imaging studies. Avendi et al. [23] trained a CNN to perform automatic segmentation of the left ventricle of the heart to calculate some clinical parameters, such as the left ventricle volume. Their results outperformed state-of-the-art methods. What is more, Gong et al. [24] report successful implementation of deep learning to reduce contrast dose by an order of magnitude in contrast-enhanced brain MRI.

### Model capabilities

As presented in Fig 3, the model was able to segment the main cerebral arteries. It can be also hypothesized that there are differences between individual examinations that cannot be seen by the model. First of all, if the model learned the anatomy of the brain blood vessels, it may predict the course of the main cerebral arteries. However, the individual variability of the small vessels might make it impossible to segment the vessels accurately in all cases. Therefore, the wide range of Dice coefficients for single patients (min = 0.467068, max = 0.720970) may not have been not due to an imperfect model, but rather due to individual differences between the cases.

On the other hand, if the model has learned to recognize blood vessels on NCCT scans in general, without specifically learning cerebral anatomy, it is possible that small vessels are too small to be recognised, taking into account the resolution of a CT scan. Thirdly, the degree of the filling of the small vessels varied between individual examinations, as shown in Fig 3, which could have also influenced the maximum achievable Dice coefficient. The ground truth masks used in the study were not perfect and sometimes some parts of the vessels were not segmented due to limitations of the segmentation method. In a few test cases, the model was nonetheless able to predict the artery that was omitted in the original mask (Fig 4). This proves a good generalization of the model’s performance but in this setting, the correct prediction lowered the Dice coefficient for the examination.

What is more, as a result of a random patient selection, one of the test cases was a CT scan with a wider than usual scanning area and a large basilar artery aneurysm (2.3 cm in diameter) in an unusual location. There is no standardized scanning range for head NCCT, as this is dependent on the pathology being examined and the scanner operator. For this reason, a wide scanning area was not an exclusion criterion in this study, but has nonetheless significantly affected the Dice results on the test set, as the model was trained on a narrower scanning range. Although aneurysms as big as 2.3 cm are not common, in our opinion, it is also not a valid exclusion criterion, as this method could someday be used to detect those aneurysms; however, the aneurysm this size affected the Dice coefficient. The lowest Dice coefficient in our test set (0.467068) is a result of prediction on the data with both a wide scanning area and a large aneurysm in an unusual location. When this patient was excluded, the mean Dice coefficient for the test set was 0.655344. In contrast, the mean Dice coefficient for the entire test set was 0.638228.

We also compared our model that benefited from a variety of the latest improvements for the neural networks with the original U-Net as proposed by Ronnenberger et al. [11]. The pretrained model had a slight advantage over the original U-Net architecture, albeit the difference was relatively small (mean Dice coefficient for cross-validation was 0.673 and 0.660, respectively).

### Registration

Image registration is frequently used for radiological image processing [25]. In the initial stages of the project, it was intended to create an original semi-automatic registration tool that pre-matched the level of CTA and NCCT scans and required manual verification by a radiologist. However, this has proven to be a time-consuming approach and the quality of the results was not satisfactory. For this reason, it has been decided to make use of an already existing tool. A large number of software solutions have been available, including 3D Slicer [26] and Elastix [27], both based on the ITK library [28]. In this case, 3D Slicer has proven to provide very accurate registrations.

### Segmentation

Segmentation of the contrasted blood vessels remains an important challenge in the field [29]. Manual segmentation is a very tedious task, and various approaches for automatic and semi-automatic segmentation have been tested [30]. Semi-automatic segmentation has proven to be the most efficient method in this case. Although the use of segmentation in radiographic imaging has been quite extensive, vessel segmentation remains a problem that is significantly more complex than, for example, the segmentation of the brain tissue [31] or ventricles. Other researchers attempting vessel segmentation have used various approaches, with a common assumption that the difference in the radiodensity measured in HU is the key to the segmentation. Generally, each approach consists of data preparation that aims to enhance the visibility of the vessels followed by pre segmentation, i.e., filtering regions of interest, and the segmentation itself [25]. The most commonly used approaches include thresholding, border-based techniques, and region growing [10]. Having attempted all of the above, it was concluded that the semi-automatic method used in this study, based on thresholding combined with the subtraction, outperformed each of these techniques. Thresholding alone could not provide us with adequate vessel segmentation, as contrasted vessels have similar radiodensity to the bone tissue. In the case of region-growing techniques, it was difficult to determine the optimal location and number of seed points, particularly when taking into account anatomical differences between patients. What is more, in the points of the proximity of the bone and vessels, a leak out of the region growing to the bone tissue was a significant problem. As to the active contour technique, it was discovered that this technique was unable to correctly isolate blood vessels in the regions where the vessels pass near the bone or have calcified atherosclerotic plaques. Thresholding the radiodensity and subtracting the obtained non-contrast mask from the contrast mask, as well as various issues concerning the above-mentioned approaches, have been described in detail by Hedblom and Unger [32].

### Dataset analysis

#### Number of patients in the dataset

**Fig 5:**
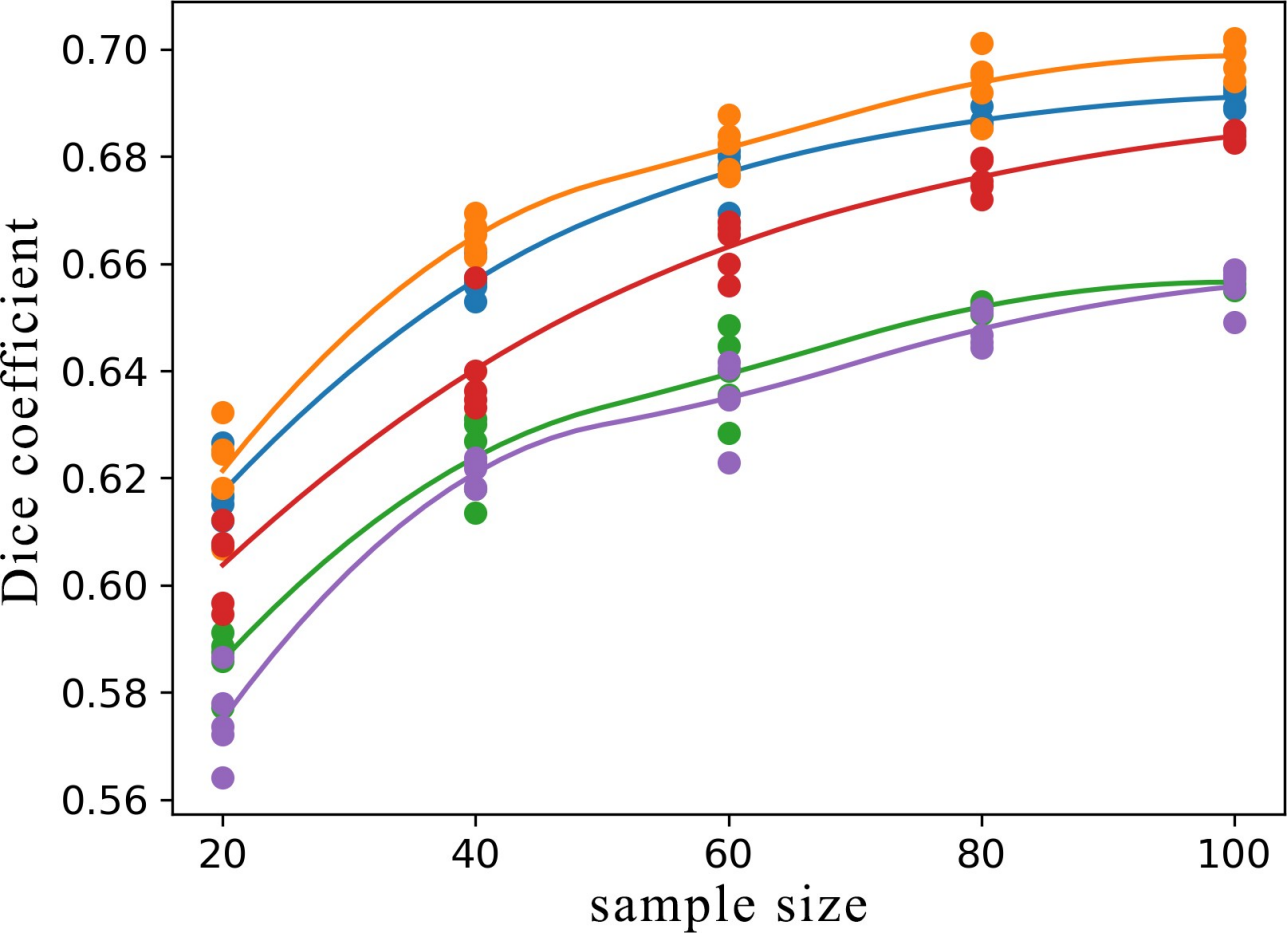
Mean Dice coefficient performance on sampled 12 patients validation sets with the training sets varying in sizes. Each point represents a metric achieved by training a model using the subsampled training set of a given size.

As a part of the evaluation, an analysis of the training set size impact on the algorithm performance was performed. Five 12-patients disjoint samples from the cross-validation dataset were isolated and used as a temporary validation set. The remaining patients were used for subsampling training sets of a smaller size. Training sets containing 20, 40, 60, 80 and 100 patients were tested. The process was repeated for each temporary validation set five times for each sample size. The improvement in performance resulting from a bigger size of the training set was measured by comparing Dice coefficients of the temporary validation set. For the training set size impact analysis, the best performing preprocessing approach was used. Fig 5 demonstrates the effect of increasing the number of patients in the training set. The small difference in performance between training sets consisting of 80 and 100 patients suggested that adding more patients would not improve the Dice coefficient significantly using our architecture of choice. The biggest gain between the sample sizes was observed between the training set sizes of 20 and 40 patients. The bigger the training set, the smaller the impact of adding patients to the training set.

#### Gender distribution in the dataset

The gender distribution of the study group does not represent the general population, because the women were twice as numerous as the men. This could potentially result in a biased model, resulting in a different performance depending on the patient's gender. To analyze this risk, the Dice coefficient of individual validation patients during cross-validation was stored and compared between genders using the Mann-Whitney U test. The p-value of 0.2805 does not allow us to reject the hypothesis that Dice coefficients of males and females are equal.

### Alternative approaches to cerebral angiography segmentation from NCCT

In the early stages of the study, other approaches to the problem were also explored.

In the process of choosing the hyperparameters for the model, a wide variety of segmentation loss functions were tested following publicly available implementations [33]; however, none of those outperformed the original generalised Dice loss function [22].

It was attempted to use CycleGAN, which is a deep learning method of image-to-image translation [34]. This seemed ideal for the problem, as a model that could learn to translate NCCT to CTA scans and vice versa could potentially be developed. The main advantage of this approach is no need for creating segmentations. However, during the training, the network seemed to be unable to generate any vessels from the NCCT. CycleGAN has a multistage loss function based on an L1 loss function. It is hypothesized that in this case, L1 loss focused the training process on increasing the mean radiodensity of the image rather than increasing only the radiodensity of the vessels. Therefore, it might be beneficial to create a new loss function that could reward the network for accurately reproducing details of the scan that made up only a small fraction of the area of each image, i.e., the vessels.

It was also attempted to use a 3D U-Net architecture [35] with the assumption that providing the network with additional information about the continuity of the vessels might yield better results. However, with limited computational resources available, no promising results were obtained which would encourage the team to pursue this approach further.

### Limitations of this study

Although the results of this research are promising, some limitations of this study were identified. It should be noted that all the examinations on which the model was tested were performed on one CT scanner. It cannot be excluded that the trained network would yield different results on scans obtained from a different scanner.

Main cerebral arteries have a relatively predictable course in relation to the skull. Theoretically, an algorithm which identifies those regions based on the bone features could provide segmentations while completely ignoring the actual presence of the vessels. To prevent this issue from occurring, a large subset of the training data consisted of patients with an intracranial aneurysm, which is much less predictable in terms of localization than intracranial arteries.

It should also be underlined that the “ground truth” masks used in this study were sometimes imperfect due to limitations of the segmentation method. Training the neural network on higher quality masks might result in an even better performing model.

## Conclusions

In summary, cerebral angiography synthesis from NCCT scans using deep learning state-of-the-art techniques has been proven to produce promising synthetic images. This research is an example of leveraging deep learning techniques to deliver new diagnostic tools for clinicians that will facilitate patient care in challenging situations. This method could be applied in a clinical setting as an aid in cases where CTA would be clinically relevant, but cannot be performed due to various contraindications. To facilitate implementation in a clinical setting in other hospitals and to encourage further research in the field, we provide free access to all the code we produced for this research [36].
